# A Cumulative Electrical Risk Score Is Associated with Appropriate ICD Therapy in Primary-Prevention Patients with Reduced Ejection Fraction: A Single-Center Retrospective Study

**DOI:** 10.3390/jcm15176829

**Published:** 2026-09-03

**Authors:** Muhammed Rıdvan Ersoysal, Rauf Avcı, Fatih Han Kumtaş, Göksel Çağırcı

**Affiliations:** 1 Department of Cardiology, Antalya Training and Research Hospital, 07100 Antalya, Türkiye; 2 Department of Cardiology, Elbistan State Hospital, 46300 Kahramanmaraş, Türkiye

**Keywords:** appropriate device therapy, electrical risk score, electrocardiography, sudden cardiac death, primary prevention, heart failure with reduced ejection fraction

## Abstract

**Background**: In heart failure with reduced ejection fraction (HFrEF), implantable cardioverter-defibrillators (ICDs) are used for the primary prevention of sudden cardiac death, yet many patients never receive appropriate therapy, and left ventricular ejection fraction (LVEF) alone poorly discriminates who will. We investigated whether a cumulative electrical risk score (ERS) derived from the surface electrocardiogram is associated with appropriate ICD therapy. **Methods**: In this single-center, retrospective study, 205 patients with HFrEF who underwent ICD implantation for primary prevention were classified according to whether they had received appropriate ICD therapy. A modified eight-parameter ERS was calculated from the baseline electrocardiogram. Associations were assessed using receiver operating characteristic (ROC) analysis and logistic regression. **Results**: The ERS showed moderate-to-good discrimination (AUC 0.795; *p* < 0.001); a cut-off of 3.5 yielded 91% sensitivity and 71.4% specificity. After adjustment for age, sex, LVEF, cardiomyopathy etiology, BNP and device duration, the ERS remained independently associated with appropriate ICD therapy (adjusted OR 2.189; 95% CI 1.246–3.845; *p* = 0.006). **Conclusions**: A cumulative electrical risk score from the standard electrocardiogram was independently associated with appropriate ICD therapy and may add risk information beyond LVEF. These retrospective findings warrant prospective, multicenter validation.

## 1. Introduction

Heart failure (HF) remains one of the leading causes of morbidity and mortality worldwide. According to current epidemiological data, approximately 56 million people are living with HF globally, and this number is expected to rise further in the coming years as the population ages [1]. A substantial proportion of deaths in patients with heart failure with reduced ejection fraction (HFrEF) is attributable to sudden cardiac death (SCD) caused by ventricular tachyarrhythmias [2].

In appropriately selected patients with HFrEF, implantable cardioverter-defibrillator (ICD) therapy is widely used for the primary prevention of SCD [3,4,5,6,7]. The EU-CERT-ICD study, conducted in European patients eligible for primary-prevention ICD therapy, demonstrated a significant 27% relative reduction in mortality between the ICD and control groups in the overall cohort, reflecting the survival benefit of ICD therapy [5]. Nevertheless, a substantial proportion of patients who receive an ICD for primary prevention never receive any appropriate device therapy (antitachycardia pacing or shock) during follow-up [8]. At the same time, these patients remain exposed to the risk of inappropriate shocks, device- or lead-related infection, and other procedural complications [8,9,10]. Therefore, better identification—before implantation—of patients who will truly benefit from ICD therapy represents an important clinical need, both to avoid unnecessary procedures and to prevent potential complications.

The electrocardiogram (ECG) is a widely used, readily accessible, non-invasive, and low-cost diagnostic tool for the diagnosis and follow-up of cardiovascular diseases. In a population-based study published in 2017, a cumulative electrical risk score (ERS) combining multiple depolarization and repolarization parameters derived from the surface ECG was shown to be independently associated with SCD and to improve risk stratification beyond the left ventricular ejection fraction [11]. However, the association of this score with appropriate ICD therapies in patients who underwent ICD implantation for HFrEF has not yet been sufficiently investigated.

In this study, we aimed to investigate the association between the ERS calculated from the surface ECG and appropriate device therapies in patients who underwent ICD implant for HFrEF. We hypothesized that a cumulative electrical score would be associated with the occurrence of appropriate ICD therapy beyond LVEF alone.

## 2. Materials and Methods

### 2.1. Study Design and Population

This single-center, retrospective study included patients who presented to the cardiac implantable electronic device (CIED) outpatient clinic of a tertiary care center between January 2025 and January 2026. Among the 504 patients evaluated during this period, those who had undergone ICD implantation for primary prevention with a diagnosis of HFrEF were screened. A total of 276 patients meeting these criteria were initially assessed.

Patient data were obtained retrospectively from the outpatient registry and the hospital information management system. Echocardiographic, laboratory, and ECG data were retrieved from the most recent follow-up parameters recorded prior to ICD implantation.

**Figure 1:**
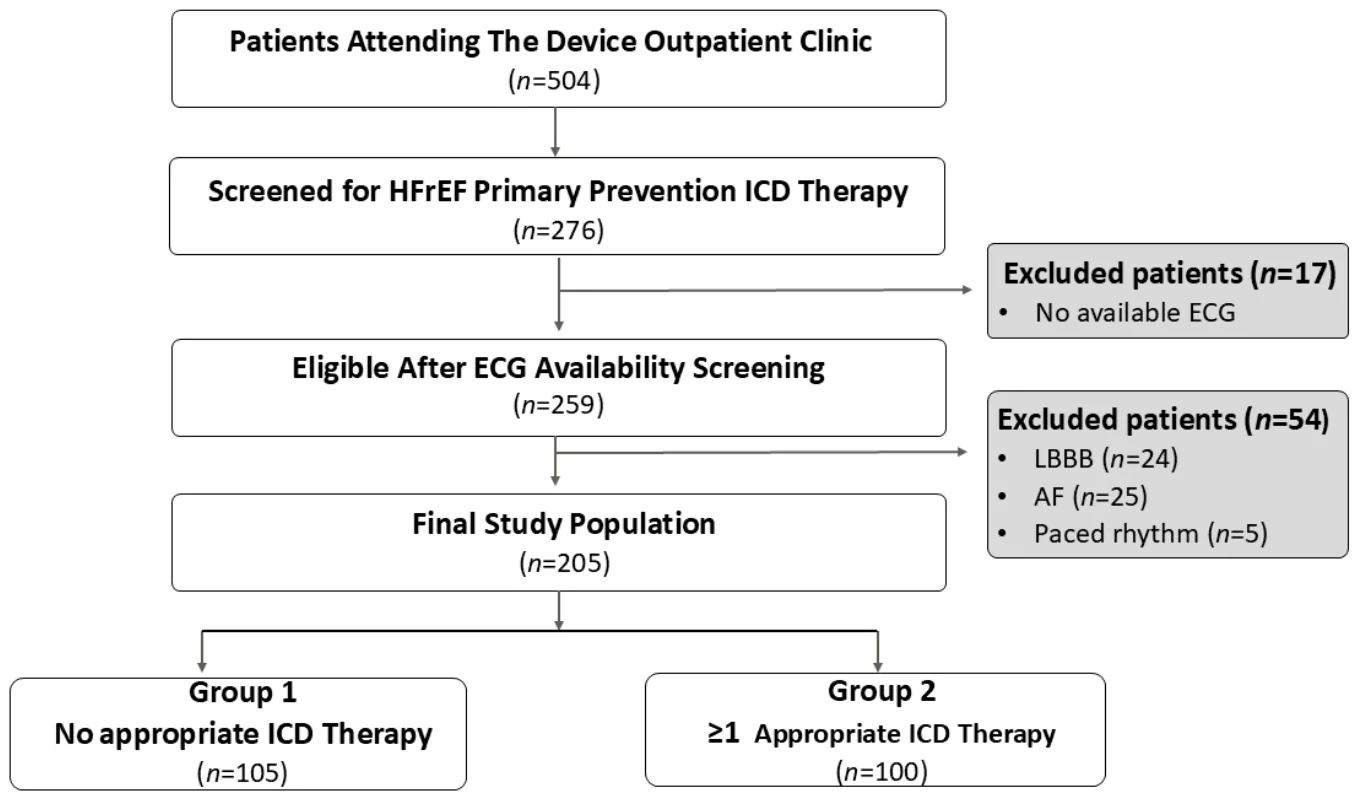
Patient selection flow chart.

Seventeen patients without an available or evaluable ECG recording in the system were excluded. As part of routine clinical practice at our center, a standard 12-lead ECG is recorded at the visit during which the decision for ICD implantation is made and the device is implanted, and this ECG is archived in the hospital information system. Because the present study was retrospective in design, we did not prospectively record any new ECGs; instead, for each patient, we retrieved from the hospital information system the ECG that had been recorded at that patient’s own ICD implantation visit, whenever such a record was available. Patients for whom no such ECG could be retrieved were excluded (*n* = 17; Figure 1). Consequently, although patients were enrolled retrospectively and had heterogeneous device durations at the time of data collection, the ECG used to calculate the ERS for each individual patient corresponded specifically to that patient’s own implantation visit and therefore preceded, by definition, any subsequent appropriate ICD therapy. Patients with ECG characteristics unsuitable for the calculation of the electrical risk score—those with atrial fibrillation (AF) or left bundle branch block (LBBB), and those whose only ECG recording in the system was obtained after ICD implantation and was in a paced rhythm (a total of 54 patients)—were excluded from the study. Consequently, all analyzed ECGs were recorded during intrinsic conduction. This ensured that all analyzed ECGs were free from pacing artifact, reflected the patient’s own ventricular conduction, and preserved the reproducibility of the measurements. After these exclusions, the final analysis was performed on 205 patients. The study flow chart is summarized in Figure 1.

### 2.2. Tachycardia Programming of ICD Devices

In all patients who received an ICD for primary prevention due to HFrEF, device programming was standardized according to previous publications to reduce unnecessary shocks [12,13,14,15]. A three-zone detection strategy was programmed, comprising a monitor zone covering rates of 170 bpm and above, a ventricular tachycardia (VT) therapy zone, and a ventricular fibrillation (VF) zone.

For detection in the VT zone, 30 of the last 40 R-R intervals were required to have a cycle length (CL) < 300 ms (>200 bpm), corresponding to a detection time of approximately 9 s before therapy. In the VF zone, episodes with a CL < 240 ms (>250 bpm) in the last 8 R-R intervals were classified as VF; the detection time in this zone was kept shorter because of potential hemodynamic instability [12,13,14,15]. ICDs from different manufacturers were used in the study population (Medtronic, Minneapolis, MN, USA; Boston Scientific, Marlborough, MA, USA; and BIOTRONIK, Berlin, Germany). Supraventricular tachycardia (SVT) discrimination algorithms were programmed on a patient-specific basis according to the manufacturer-specific algorithms (Medtronic PR Logic, Boston Scientific Rhythm ID, BIOTRONIK SMART) of each device [16].

Rhythms in the range of 170–200 bpm were monitored in the monitor zone, in which no therapy (ATP or shock) was delivered. In the VT zone, therapy was programmed as two antitachycardia pacing (ATP) bursts of 8 pulses at 88% of the VT CL following a 9-s delay, followed by up to five maximal-energy shocks if the tachycardia did not terminate [13]. In the VF zone, ATP during charging was delivered following a 2.5-s delay, followed by five maximal-energy shocks.

### 2.3. Assessment of ICD Therapies

The ICD interrogation records of all included patients were reviewed. Device records were routinely retrieved using the manufacturers’ programmers and were interpreted by two cardiologists experienced in CIEDs. All therapies detected in the ICD records were classified as appropriate or inappropriate by consensus. Appropriate therapy was defined as therapy delivered in response to confirmed ventricular tachycardia or ventricular fibrillation. Patients were divided into two groups according to whether they had received appropriate ICD therapy (ATP and/or shock) during follow-up. Group 1 (*n* = 105) comprised patients who received no appropriate ICD therapy, and Group 2 (*n* = 100) comprised patients who received at least one appropriate ICD therapy. This classification was based on the presence or absence of appropriate therapy; inappropriate therapy was not the subject of comparison in this study.

Multiple appropriate ICD therapy events were evaluated using a 24-h time window: ICD therapies occurring within the same 24-h period were considered a single event, whereas therapies occurring in different 24-h periods were counted as separate events. This approach was adopted to avoid counting consecutive therapies related to the same arrhythmic episode (e.g., repeated ATP attempts or shocks) as multiple distinct events.

### 2.4. Electrocardiographic Analysis

ECG recordings were obtained using a digital electrocardiography device available at the hospital (CARDIOVIT AT-102 G2, SCHILLER AG, Baar, Switzerland). All ECGs were recorded as 10-s simultaneous 12-lead acquisitions at a paper speed of 25 mm/s and a calibration of 10 mm/mV, and the recordings uploaded to the hospital PACS system were downloaded for analysis. The recordings were processed with the device’s integrated automatic measurement and interpretation software (SCHILLER ETM Interpretation Software version 0A.106000; SCHILLER AG, Baar, Switzerland) and subsequently verified manually by cardiologists.

The PR interval, heart rate, QRS duration, QRS axis, and T-wave axis were obtained from the automated ECG reports. Other ECG measurements were performed by two cardiologists blinded to the patient groups using software that enables measurement with electronic calipers on digital ECG recordings (EP Calipers 3, EP Studios Inc., Louisville, KY, USA). Before measurement, the calipers were calibrated on each ECG using a known time interval (1000 ms) to ensure the accuracy of scaling. Intervals and heart rate were measured with time calipers, and wave amplitudes were measured with amplitude calipers.

### 2.5. Calculation of the Electrical Risk Score

The electrical risk score (ERS) was adapted from the cumulative ECG risk score defined by Aro et al. [11]. Aro et al. initially evaluated eight electrocardiographic parameters but excluded QRS duration and delayed intrinsicoid deflection from the final six-parameter score, as these were not significant in the multivariable model [11]. In our study, considering that these two depolarization markers may reflect conduction delay and the burden of myocardial scar/fibrosis in the HFrEF population, all eight parameters were retained, and the score was calculated in the range of 0–8 by assigning 1 point to each abnormal parameter.

The score components and abnormality thresholds were defined as follows. A heart rate >75 bpm and a QRS duration > 10 ms were considered elevated/prolonged. The planar frontal QRS-T angle was calculated as the absolute angle between the QRS axis and the T-wave axis, and a value >90° was considered abnormal. Left ventricular hypertrophy (LVH) was defined according to the Sokolow-Lyon (SV1 + RV5 or RV6 [whichever was greater] ≥ 35 mm) or Cornell criteria (RaVL + SV3 > 20 mm in women and >28 mm in men). The QRS transition zone was determined by identifying the precordial lead in which the R-wave amplitude was equal to or greater than the S-wave amplitude; QRS transition occurring at lead V5 or later was considered delayed. A duration ≥50 ms from the onset of the QRS to the peak of the R wave in leads V5–V6 was considered delayed intrinsicoid deflection.

**Figure 2:**
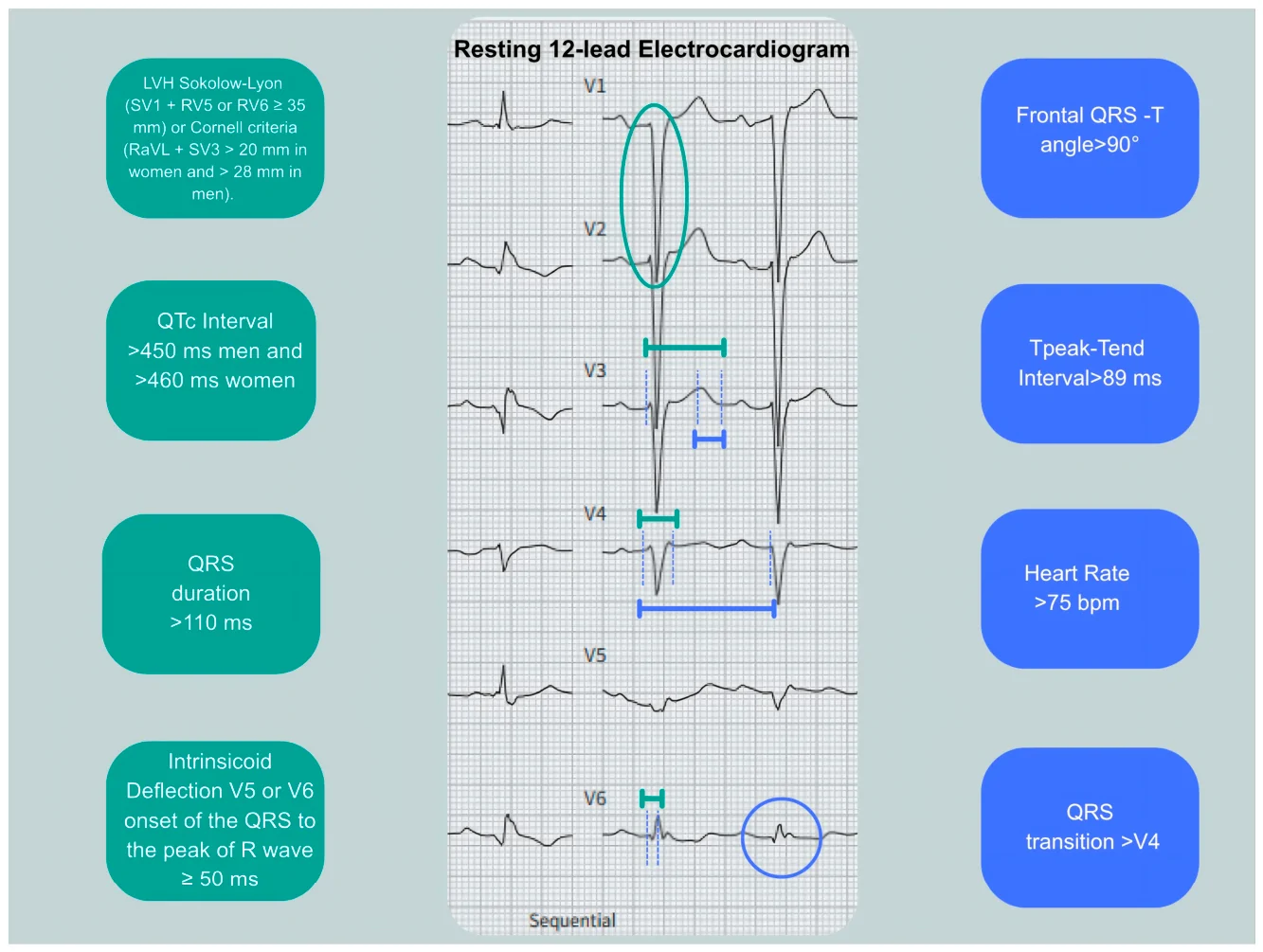
Schematic representation of the eight electrocardiographic components of the electrical risk score (ERS) on a representative surface ECG tracing. The components include heart rate (>75 bpm), QRS duration (>110 ms), frontal QRS-T angle (>90°), left ventricular hypertrophy (Sokolow-Lyon or Cornell criteria), delayed intrinsicoid deflection (≥50 ms in leads V5–V6), delayed QRS transition (at or after V5), prolonged corrected QT interval (QTc; Bazett > 450 ms in men, >460 ms in women), and prolonged Tpeak–Tend (Tp-e) interval (>89 ms). Each component present contributes one point to the cumulative ERS (range 0–8). Dashed lines indicate the start or end boundaries of the reference points used for measurement. In the vector diagram, blue lines correspond to the measurement range for the criterion indicated in the right-hand text box on the same row, and green lines correspond to the measurement range for the criterion indicated in the left-hand text box on the same row. The green circle marks SV1; the blue circle indicates that QRS transition occurs at V6.

The QT and Tpeak-Tend (Tp-e) intervals were measured manually using the same software (EP Calipers 3, EP Studios Inc., Louisville, KY, USA). The longest QT interval among all precordial and limb leads was recorded; the QT interval was corrected for heart rate using the Bazett formula to obtain the QTc, and a QTc > 450 ms in men and >460 ms in women was considered prolonged. The Tp-e interval was measured from lead V5 whenever possible, and a Tp-e interval >89 ms was considered prolonged, in line with the threshold defined in the reference study [11]. A schematic representation of the eight ERS components on a representative ECG tracing, along with their respective abnormality thresholds, is provided in Figure 2. All ECG measurements and outcome classifications were performed jointly by two cardiologists blinded to patient outcomes, with final values determined by consensus.

The HF treatment was assessed according to the 2021 ESC HF guidelines and the 2023 focused update. Patients receiving at least three of the four foundational class I–recommended drug classes in HFrEF—angiotensin receptor–neprilysin inhibitor (ARNi) or angiotensin-converting enzyme inhibitor/angiotensin receptor blocker, beta-blocker, mineralocorticoid receptor antagonist (MRA), and sodium-glucose cotransporter-2 (SGLT2) inhibitor—at the time of ICD implantation were considered to be on guideline-directed medical therapy (GDMT) [17,18].

### 2.6. Statistical Analysis

Data were analyzed using SPSS version 27 (IBM Corp., Armonk, NY, USA). Categorical data were expressed as percentages and frequencies. The distribution of numerical data was assessed using the Kolmogorov–Smirnov test. Normally distributed data were presented as mean ± standard deviation, and non-normally distributed data as median (interquartile range). Categorical variables were compared using the chi-square test. Student’s *t*-test was used for normally distributed numerical variables, and the Mann–Whitney U test for non-normally distributed variables. Missing data were handled using complete-case analysis; each variable was analyzed based on the patients for whom data were available.

Variables that were statistically significant in the between-group comparisons were evaluated using univariable logistic regression analysis. Variables that were statistically significant in the univariable analysis, together with those considered clinically relevant, were included in the multivariable logistic regression model. We acknowledge that selecting covariates on the basis of univariable statistical significance, combined with clinical relevance, is a pragmatic and widely used approach in exploratory clinical research, but it may introduce model-selection bias and can inflate the apparent significance of the retained variables. This approach was chosen given the modest sample size, which precluded a more data-intensive selection method (e.g., penalized regression); nonetheless, this constitutes an additional reason for interpreting the reported associations, including that of the ERS, with appropriate caution. Given that including the composite ERS and the individual ECG components constituting the score in the same model could lead to multicollinearity, the ECG sub-parameters and the cumulative ERS were evaluated in separate multivariable models. The results of the logistic regression analyses were presented as odds ratios, 95% confidence intervals, and *p*-values.

The receiver operating characteristic (ROC) curve was used to evaluate the association between the ERS (independent variable) and the dependent variable (appropriate ICD therapy), and the cut-off value was determined using the Youden index. Logistic regression analysis was performed to assess the effect of the independent variables on the dependent variable.

To assess the risk of overfitting and to confirm the generalizability of the model, internal validation was performed using 10-fold cross-validation. In addition, to test the stability of model performance, a 95% confidence interval for the relevant performance metric was calculated using bootstrap resampling with 2000 replications. Internal validation analyses were performed using R (version 4.6.1; R Foundation for Statistical Computing, Vienna, Austria). A *p*-value < 0.05 was considered statistically significant.

## 3. Results

### 3.1. Study Population and Baseline Characteristics

Patients were divided into two groups according to their prior appropriate ICD therapy status. The 105 patients who received no appropriate therapy during follow-up constituted Group 1, and the 100 patients who received at least one appropriate ICD therapy constituted Group 2 (total *n* = 205). All patients were in sinus rhythm. Age was similar between the two groups (63.52 ± 11.13 vs. 62.61 ± 12.84 years for Group 1 and Group 2, respectively; *p* = 0.586). Of the 100 patients in Group 2, 70 received appropriate therapy only once, whereas multiple appropriate ICD therapies were recorded in 30 patients. Device duration was significantly longer in Group 2 (median 72 vs. 36 months; *p* < 0.001). The demographic and clinical characteristics of the patients are presented in Table 1.

### 3.2. Laboratory Findings

No significant differences were found between the groups in creatinine, GFR, sodium, potassium, total cholesterol, HDL, LDL, CRP, white blood cell count, hemoglobin, platelet count, neutrophil count, lymphocyte count, troponin, or TSH levels (all *p* > 0.05). Calcium level was slightly higher in Group 1 (9.3 vs. 9.1 mg/dL; *p* = 0.029), and albumin level was also higher in Group 1 (43.2 ± 3.7 vs. 42.8 ± 2.8 g/dL; *p* = 0.005). In contrast, triglyceride level was lower in Group 2 (112.47 ± 52.68 vs. 171.88 ± 183.83 mg/dL; *p* = 0.013). The B-type natriuretic peptide (BNP) level was significantly higher in patients who received appropriate ICD therapy (median 3587 vs. 479 pg/mL; *p* = 0.034). The comparison of biochemical parameters is presented in Table 2.

### 3.3. Association Between the Electrical Risk Score and Appropriate ICD Therapy

**Figure 3:**
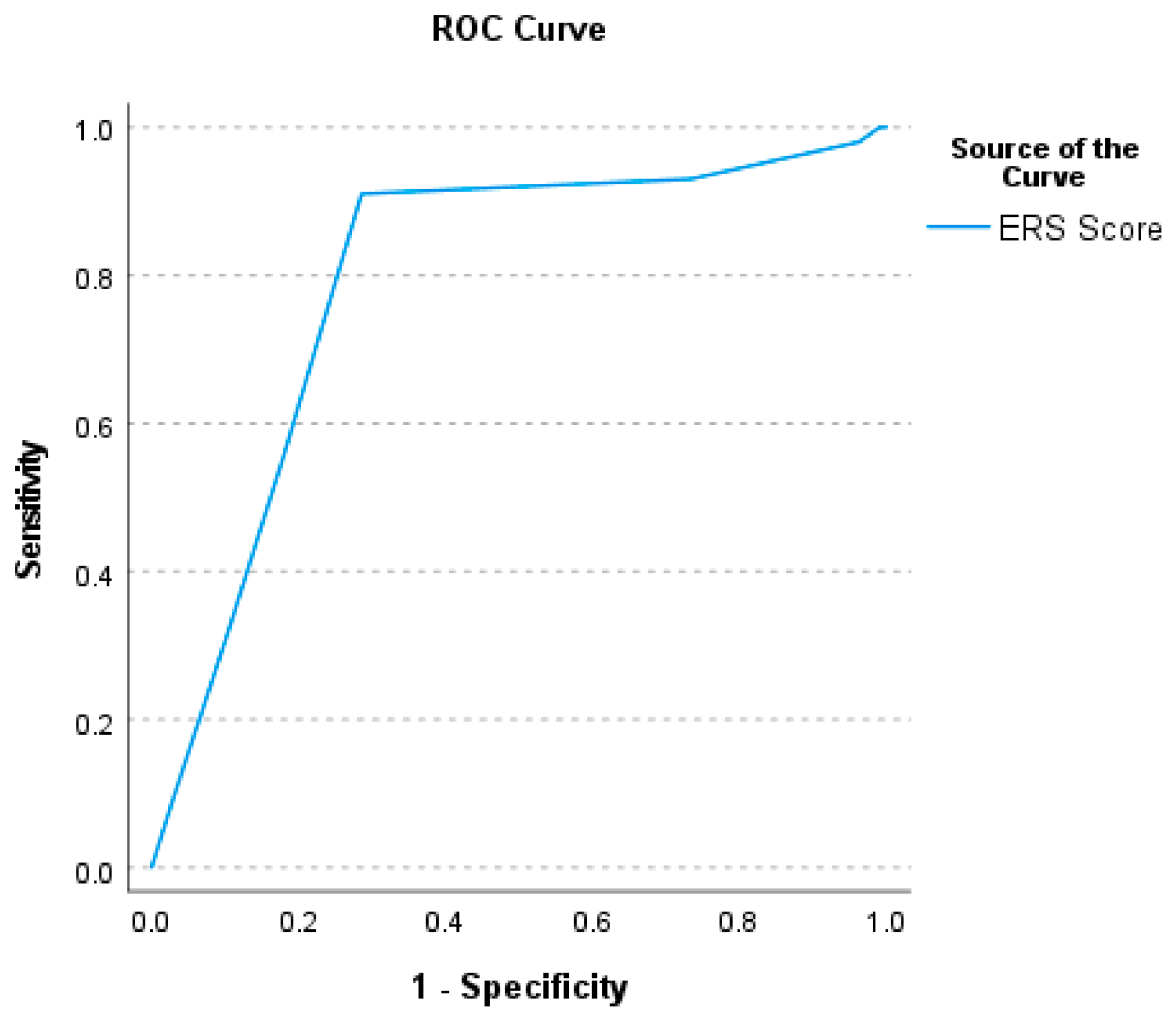
Receiver operating characteristic (ROC) curve for the electrical risk score in discriminating patients with appropriate ICD therapy (AUC = 0.795).

In the ROC curve analysis, the area under the curve of the ERS for discriminating patients who received appropriate ICD therapy was 0.795 (*p* < 0.001). The optimal cut-off value was determined to be 3.5, providing a sensitivity of 91% and a specificity of 71.4%. Because the score consists only of integers, a clinically applicable threshold of ERS ≥ 4 was adopted (Figure 3, Table 3). The potential determinants of appropriate ICD therapy were evaluated using univariable and multivariable logistic regression models (Table 4). In the univariable analysis, the ERS (OR 2.350; 95% CI 1.423–3.881; *p* < 0.001) and LVEF (OR 0.899; 95% CI 0.809–0.998; *p* = 0.047) were significantly associated with appropriate ICD therapy, whereas age, sex, device duration, cardiomyopathy etiology, and BNP were not significant. In the multivariable model adjusted for age, sex, LVEF, cardiomyopathy etiology, BNP, and device duration, the ERS remained independently associated with appropriate ICD therapy (adjusted OR 2.189; 95% CI 1.246–3.845; *p* = 0.006). This indicates that each one-point increase in the ERS was associated with approximately twofold higher odds of appropriate ICD therapy. The predictive value of LVEF lost its significance after multivariable adjustment (adjusted OR 0.950; *p* = 0.471). Although LVEF as a continuous variable was significantly associated with appropriate ICD therapy in univariable logistic regression (OR 0.899; 95% CI 0.809–0.998; *p* = 0.047), the mean LVEF did not differ significantly between the two groups (30.44 ± 7.30% vs. 29.79 ± 8.91%; *p* = 0.574; Table 1), suggesting that LVEF alone provides limited discrimination at the individual level.

### 3.4. Analysis of the Electrical Risk Score Components

The association of the eight electrocardiographic components constituting the ERS with appropriate ICD therapy was also evaluated (Table 5). In the univariable analysis, most components were significantly associated with appropriate ICD therapy: delayed intrinsicoid deflection (OR 5.98; 95% CI 2.51–16.6; *p* < 0.001), prolonged Tp-e (OR 3.77; 95% CI 1.79–8.57; *p* < 0.001), delayed QRS transition (OR 3.38; 95% CI 1.89–6.16; *p* < 0.001), prolonged QRS duration (OR 3.27; 95% CI 1.86–5.85; *p* < 0.001), prolonged QTc (OR 2.67; 95% CI 1.49–4.85; *p* = 0.001), and wide QRS-T angle (OR 1.85; 95% CI 1.06–3.23; *p* = 0.03) were significant, whereas elevated heart rate (*p* = 0.59) and LVH (*p* = 0.059) were not significant in the univariable analysis.

In the multivariable model, delayed QRS transition (adjusted OR 4.33; 95% CI 2.17–9.01; *p* < 0.001), prolonged QRS duration (adjusted OR 2.21; 95% CI 1.09–4.53; *p* = 0.029), and prolonged QTc (adjusted OR 2.08; 95% CI 1.02–4.29; *p* = 0.045) retained their independent associations. Although LVH appeared significant in the multivariable analysis (adjusted OR 7.93; 95% CI 2.03–40.1; *p* = 0.006), this finding should be interpreted with caution because of its very wide confidence interval. The adjusted estimate for delayed intrinsicoid deflection could not be calculated because the model did not converge.

### 3.5. Internal Validation, Calibration and Optimism Correction

To assess model overfitting and internal validity, we performed 10-fold cross-validation and bootstrap resampling with 2000 replications. The multivariable model demonstrated strong discriminative ability, with an initial apparent AUC of 0.840. Following bootstrap resampling, the estimated model optimism was minimal (0.019), yielding an optimism-corrected AUC of 0.821 (95% bootstrap CI: 0.790–0.899). Similarly, 10-fold cross-validation confirmed consistent performance, with a cross-validated mean AUC of 0.822 ± 0.103. Model calibration, evaluated using the Brier score, showed acceptable overall accuracy, with an apparent Brier score of 0.162 and an optimism-corrected Brier score of 0.176. For the standalone ERS, the optimism-corrected AUC was 0.795 (95% bootstrap CI: 0.733–0.857) (Table 6).

**Figure 4:**
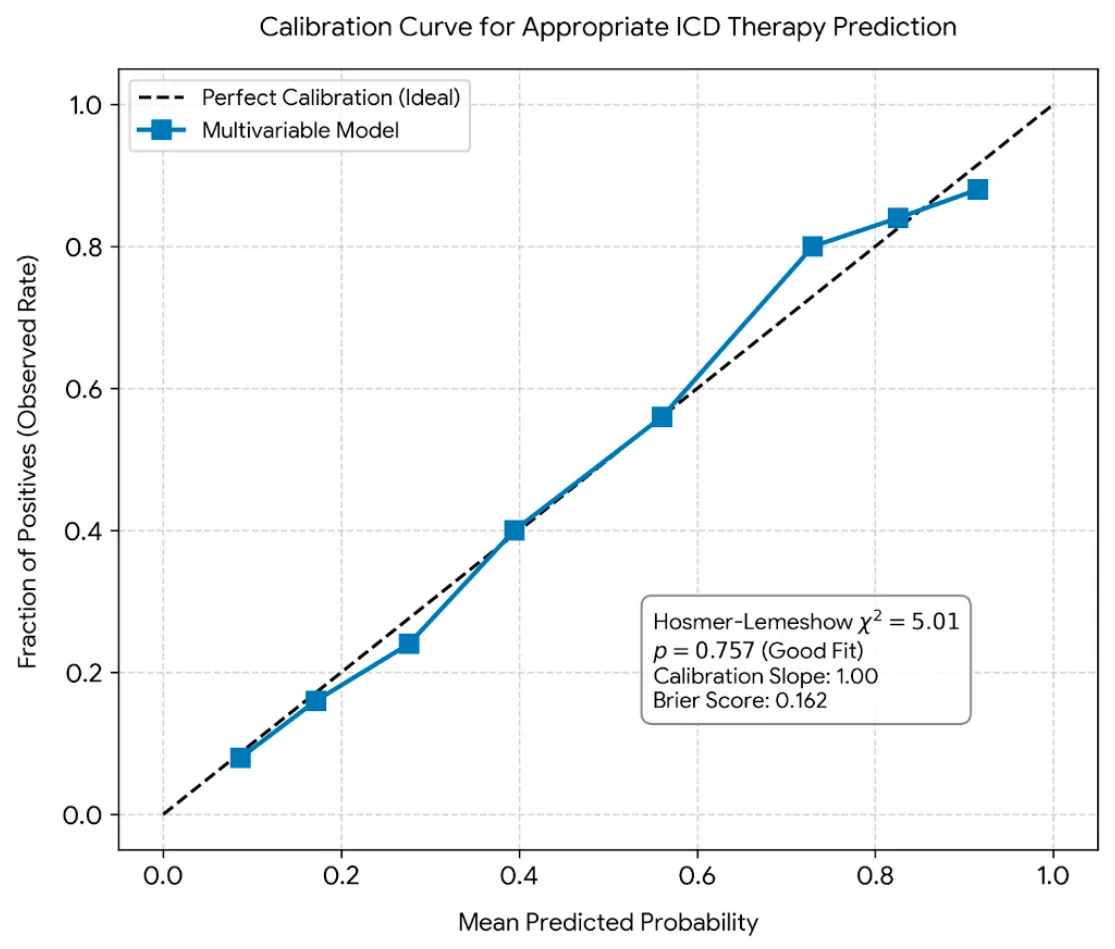
Calibration plot of the multivariable predictive model for appropriate ICD therapy. Additionally, the Hosmer–Lemeshow goodness-of-fit test and the Brier score were used as supplementary calibration measures. The dashed line represents perfect calibration (predicted probability equals observed proportion); the solid line represents the observed calibration of the model across risk deciles. The model showed excellent calibration (Hosmer–Lemeshow χ^2^ = 5.01, df = 8, *p* = 0.757; calibration slope = 1.00; Brier score = 0.162, optimism-corrected Brier score = 0.176).

To assess model calibration beyond discrimination (AUC), the Hosmer–Lemeshow goodness-of-fit test, Brier score, and calibration plots were evaluated for the multivariable model. The Hosmer–Lemeshow test demonstrated excellent agreement between predicted probabilities and observed ICD therapy events (χ^2^ = 5.01, df = 8, *p* = 0.757), indicating no significant deviation from perfect calibration. Overall model calibration was further confirmed by a Brier score of 0.162 (optimism-corrected: 0.176; scaled Brier skill score: 0.351), a calibration slope of 1.00, and a calibration intercept of 0.00. Visual inspection of the calibration curve (Figure 4) confirmed that the predicted probabilities closely aligned with the observed event rates across all risk deciles.

### 3.6. Comparison of the Modified Eight-Component ERS with the Original Six-Component Score

The modified eight-component ERS, which incorporates two additional depolarization parameters (QRS duration > 110 ms and delayed intrinsicoid deflection), was directly compared with the original six-component ERS.

The eight-component ERS demonstrated significantly better discriminative performance in this cohort compared with the six-component score for identifying patients with appropriate ICD therapy compared with the six-component score (AUC: 0.795 [95% CI: 0.733–0.857] vs. 0.731 [95% CI: 0.665–0.798]; DeLong test Z = 3.25, *p* = 0.012). Furthermore, the addition of the two depolarization markers significantly improved risk reclassification and model fit, yielding a continuous Net Reclassification Improvement (NRI) of 0.629 (*p* < 0.001), an Integrated Discrimination Improvement (IDI) of 0.056 (*p* = 0.002), and a lower Akaike Information Criterion (AIC: 193.39 vs. 199.25). In multivariable logistic regression adjusted for age, sex, LVEF, etiology, and device duration, both scores remained independently associated with appropriate ICD therapy; however, the eight-component score provided a stronger overall model fit.

## 4. Discussion

In this study, we evaluated the association between the cumulative ERS calculated from the surface ECG and appropriate ICD therapy in patients who received an ICD for primary prevention due to HFrEF. The ERS showed moderate-to-good discrimination in patients who received appropriate ICD therapy. (AUC 0.795; *p* < 0.001). In the ROC analysis, a cut-off of 3.5 provided a sensitivity of 91% and a specificity of 71.4%; because the score consists only of integers, a clinically applicable threshold of ERS ≥ 4 was adopted. After adjustment for age, sex, left ventricular ejection fraction, cardiomyopathy etiology, and BNP level, the ERS remained independently associated with appropriate ICD therapy (adjusted OR 2.19; 95% CI 1.25–3.85; *p* = 0.006). Each one-point increase in the ERS was associated with an approximately two-fold increase in the likelihood of appropriate ICD therapy.

These findings suggest that a cumulative electrocardiographic score jointly reflecting different components of cardiac depolarization and repolarization may provide additional information for assessing arrhythmic susceptibility in patients with reduced LVEF who underwent ICD implantation for primary prevention. The fact that the ERS retained its significance independently of conventional clinical variables indicates that the combined evaluation of multiple electrical abnormalities detected on the surface ECG may contribute to the stratification of the risk associated with appropriate ICD therapy.

Numerous clinical, laboratory, and electrocardiographic parameters influencing the occurrence of appropriate ICD therapy have been described in patients receiving an ICD for HFrEF or other indications. At the clinical level, reduced LVEF [19], advanced New York Heart Association (NYHA) functional class [20], older age, and non-use of beta-blockers [21] have been associated with appropriate therapy; in cohorts with coronary artery disease, older age and reduced LVEF have been reported as independent predictors [22]. Similarly, elevated BNP levels and impaired renal function have been associated with an increased risk of ventricular arrhythmia and appropriate ICD therapy [23,24]. Nevertheless, the discriminative value of these variables may vary across study populations and may be limited in selected cohorts in which all patients received an ICD because of low LVEF. In our study, no significant difference in LVEF was found between the groups (30.44 ± 7.30% vs. 29.79 ± 8.91%; *p* = 0.574), further supporting the notion that LVEF alone provides limited discrimination between patients who do and do not receive appropriate ICD therapy. Similarly, although the BNP level differed between the groups in our cohort, it was not independently associated with appropriate ICD therapy in the multivariable analysis. However, because all parameters were obtained only from the values available at the visit during which ICD implantation was performed, BNP values were missing in a considerable number of patients. Since this missing data could introduce bias into the interpretation of the BNP findings, these results should be evaluated with caution. The lack of difference in renal function between the groups also indicates that conventional clinical variables alone may be insufficient to discriminate patients who receive appropriate ICD therapy in this selected population.

The standard surface ECG is an important clinical tool for assessing the risk of ventricular arrhythmia because it is low-cost, non-invasive, and widely accessible [25]. Electrocardiographic markers such as prolonged QTc, increased Tp-e interval, wide frontal QRS-T angle, delayed intrinsicoid deflection, and delayed QRS transition have been reported to reflect repolarization heterogeneity, intraventricular conduction delay, and electrical disarray associated with myocardial scar or fibrosis [26,27,28,29]. Indeed, in our cohort as well, most individual markers did not retain independent associations in the multivariable model, whereas the cumulative ERS remained independently associated with appropriate ICD therapy; when we directly compared the modified eight-component ERS with the original six-component score, the eight-component score demonstrated significantly better discrimination, reclassification, and model fit (Table 7), further supporting the value of combining multiple electrical abnormalities within a single cumulative measure. Therefore, the combined evaluation of multiple ECG abnormalities reflecting different aspects of depolarization and repolarization within a cumulative score may allow a more comprehensive characterization of the arrhythmogenic substrate.

In the population-based Oregon SUDS and ARIC cohorts, Aro et al. combined electrocardiographic abnormalities reflecting different aspects of cardiac depolarization and repolarization into a cumulative score and demonstrated that an increasing burden of electrical abnormalities was associated with the risk of sudden cardiac death [11]. Our study evaluated this approach in a more selected and a relatively homogeneous clinical population, in which all patients had received an ICD for primary prevention due to HFrEF. Whereas the final ERS model of Aro et al. comprised six components, our study additionally retained QRS duration and delayed intrinsicoid deflection to more comprehensively reflect depolarization delay in the HFrEF population, using a modified eight-component ERS [11].

Important differences exist between the two studies in terms of patient population, design, and endpoint. Whereas the study by Aro et al. assessed sudden cardiac death in community-based populations, our study was based on appropriate ICD therapy verified through device records and intracardiac electrograms [11]. The direct recording of ventricular arrhythmia episodes by the device allows objective classification of the endpoint. Nevertheless, appropriate ICD therapy is not equivalent to sudden cardiac death or to the survival benefit derived from an ICD. Therefore, although a direct comparison of effect sizes between the two studies is not appropriate, the finding that the cumulative burden of electrical abnormalities is associated with arrhythmic endpoints across different clinical contexts suggests that the ERS may be a marker capable of providing additional information about the underlying arrhythmogenic predisposition.

Our study has several original and strong aspects. To our knowledge, this is one of the few studies to evaluate the cumulative electrical risk score in a clinically selected population implanted with an ICD for primary prevention due to HFrEF. Whereas previous studies mainly examined the ERS in the context of sudden cardiac death in community-based cohorts, our study extends this approach to a mechanistically related but distinct clinical endpoint—appropriate ICD therapy verified by device records. In this respect, the study provides new data on the potential value of the cumulative burden of electrical abnormalities in a selected primary prevention ICD population.

Another original aspect of the study is the retention of QRS duration and delayed intrinsicoid deflection, in addition to the electrocardiographic components of the original ERS, to more comprehensively reflect depolarization delay and possible myocardial structural abnormality in the HFrEF population, and the evaluation of a modified eight-component score. This approach allowed the integration of abnormalities in different phases of depolarization and repolarization within a single cumulative measure. When directly compared, the modified eight-component ERS demonstrated significantly better discrimination, reclassification, and model fit than the original six-component ERS in the present cohort (Table 7); nevertheless, because this comparison was performed within a single, retrospective dataset, studies directly comparing the two scores in independent external cohorts are warranted.

The classification of appropriate ICD therapies by two cardiologists experienced in CIEDs, based on device records and intracardiac electrograms and reached by consensus, increased the objectivity of endpoint assessment and reduced the bias that may arise in classifications based on patient self-report or diagnostic codes. The joint evaluation of the electrocardiographic measurements by two cardiologists blinded to patient outcomes, with final values determined by consensus, further strengthened the consistency of the measurement process. In addition, the fact that the ERS components can be obtained from the standard 12-lead surface ECG using low-cost, non-invasive, and widely accessible methods supports the potential clinical applicability of the score.

In the three-year follow-up of MADIT II, the rate of appropriate ICD therapy was reported as approximately 35%, and in the five-year follow-up of SCD-HeFT as approximately 21% [3,13]. In our study, the rate of appropriate ICD therapy (49%; 100/205) was higher than in these studies. However, because these were large, multicenter randomized controlled trials, a direct comparison of rates with our single-center, retrospective design is limited. This higher observed rate may be related to the fact that the cohort consisted of prevalent cases attending the device clinic and that the center where the study was conducted is a referral clinic for device therapy in the region. In particular, the more frequent presentation of patients who had previously experienced ICD therapy for follow-up may have contributed to the overrepresentation of these patients in the study population and to the observed event rate being higher than in general primary prevention ICD populations.

Cardiac magnetic resonance imaging (MRI) has been shown to strongly associate myocardial scar/fibrosis detected by late gadolinium enhancement with ventricular arrhythmia and sudden cardiac death, providing additional risk stratification information beyond the left ventricular ejection fraction [30]. However, because cardiac MRI is a relatively expensive and time-consuming modality that—as in many centers—was not widely available in routine clinical use at the center where our study was conducted, a direct comparison with the ERS could not be performed. In contrast, because the ERS can be obtained inexpensively and widely from the standard surface ECG, it may hold value as a complementary risk marker in settings where access to cardiac MRI is limited.

The high sensitivity of the ERS ≥ 4 threshold may be regarded as a feature that could be useful for identifying patients with a higher likelihood of receiving appropriate ICD therapy. However, this threshold was derived from the present study population and has not been validated in an independent external cohort. The fact that the ERS, as a continuous variable, retained its independent association with appropriate ICD therapy suggests that the score may be considered not only as a categorical threshold but also as a graded risk indicator reflecting an increasing burden of electrical abnormalities. Nevertheless, our study was not designed to evaluate the use of the ERS in decisions regarding ICD implantation or generator replacement. Therefore, the ERS should be regarded not as a tool replacing current guideline-based decision processes, but as a complementary marker that may contribute to future multivariable risk stratification models.

Because permanent devices carry non-negligible risks, such as device infection (occurring in approximately 1–2% of implants), improving patient selection for primary-prevention ICD implantation is also clinically important [31,32]. As many primary-prevention patients—including a considerable proportion of our own cohort—never receive appropriate therapy while remaining exposed to these risks, risk scores such as the ERS may eventually contribute to evaluating this balance. However, the present study did not directly assess ICD implantation decisions, and this application remains speculative at this stage.

Another potential use of the ERS may lie not in the decision to implant a permanent ICD, but in identifying higher-risk patients suitable for monitoring with a wearable cardioverter-defibrillator (WCD) during the bridging period before permanent implantation; indeed, appropriate patient selection has been shown to be closely linked to the benefit derived from WCD use [33,34]. However, our study did not evaluate WCD use or outcomes, and this application likewise remains hypothetical.

### Limitations

This study has several limitations. First, the single-center, retrospective design limits causal inference and the generalizability of the findings to different clinical settings. Second, the relatively limited sample size made it difficult to reliably assess subgroup analyses and possible interactions in particular. Third, appropriate ICD therapy is not a validated surrogate endpoint for sudden cardiac death or for the survival benefit derived from an ICD; some ventricular arrhythmias treated by the device may represent episodes that could have terminated spontaneously or might not have been fatal. The relatively low rate of SGLT2 inhibitor use in our cohort may reflect the recent adoption of this drug class in our country, such that not all eligible patients had yet been reached during the study period.

The composition of the study population from prevalent patients presenting to the device outpatient clinic may also have introduced selection bias. Because the center where the study was conducted is a referral clinic for device therapy in the region, and because patients frequently presented to the center not only for routine follow-up but also after ICD therapy, patients who had previously received appropriate ICD therapy may have been overrepresented in the cohort. This may have contributed to the higher rate of appropriate ICD therapy in our study compared with previous primary prevention cohorts.

Notably, device duration was longer in patients who received appropriate ICD therapy; to partially account for this potential confounder, device duration was included in the multivariable model. However, the duration since the diagnosis of heart failure or cardiomyopathy could not be reliably retrieved from the records in a considerable proportion of patients because of the retrospective design of the study and could therefore not be included in the multivariable model. Because a longer history of cardiomyopathy may influence both electrical remodeling and the likelihood of ventricular arrhythmia, the inability to assess disease duration constitutes a limitation, and the findings should be interpreted with caution in this context.

In addition, because the analyses were performed using logistic regression based on the presence of appropriate ICD therapy, they do not fully account for differences in follow-up duration between patients or for the time to first appropriate therapy. Reconstructing event dates retrospectively was not feasible without recalling patients for a dedicated verification visit, which was beyond the scope of the present study and would have introduced additional selection bias due to incomplete attendance. Therefore, the results should be interpreted within the framework of an independent association with a history of appropriate ICD therapy rather than a time-dependent prognostic relationship. The exclusion of patients in whom the ERS could not be reliably calculated because of AF, LBBB, or paced rhythm also limits the applicability of the results to the general HFrEF and ICD population. Finally, although the modified eight-component ERS demonstrated significantly better discrimination, reclassification, and model fit compared with the original six-component ERS in the present cohort (Table 7), this comparison was performed within a single, retrospective dataset and has not been confirmed in an independent external cohort. It is important to note that internal validation, including cross-validation and bootstrap optimism correction, quantifies and partially corrects for overfitting within the same dataset from which the model was derived, but it does not substitute for external validation in an independent cohort; the internally validated performance metrics reported here should therefore not be interpreted as evidence of generalizability beyond the present study population. Furthermore, given the relatively small sample size and the number of variables included in our multivariable models, the component-level analysis (Table 5) should be regarded as exploratory rather than confirmatory; some component-level estimates should be interpreted with caution; for example, the wide confidence interval observed for left ventricular hypertrophy (adjusted OR 7.93; 95% CI: 2.03–40.1) reflects the small number of events in this subgroup. Taken together, the findings need to be confirmed in larger, multicenter, prospective, and externally validated studies employing survival analyses, such as Cox proportional hazards models with time to first appropriate ICD therapy as the endpoint, to obtain more stable effect estimates and to better account for differing observation times.

## 5. Conclusions

The modified cumulative electrical risk score calculated from the standard surface ECG was independently associated with appropriate ICD therapy in patients with reduced ejection fraction implanted with an ICD for primary prevention. These findings suggest that the ERS may be a low-cost and non-invasive marker capable of providing additional risk information beyond conventional clinical variables. Nevertheless, the prognostic value of the ERS and its potential contribution to clinical decision-making need to be investigated in multicenter, prospective, and externally validated studies that jointly evaluate electrical, structural, and clinical risk markers.

## Figures and Tables

**Table 1:** Demographic, clinical, echocardiographic, and electrocardiographic characteristics of the two groups.

Variable	Group 1 (*n* = 105)	Group 2 (*n* = 100)	*p*-Value
Female, *n* (%)	18 (17.1)	12 (12.0)	0.298
Age, years	63.52 ± 11.13	62.61 ± 12.84	0.586 ^a^
Device type, *n* (%)			0.059 ^c^
Single-chamber (VR)	98 (93.3)	85 (85.0)	
Dual-chamber (DR)	6 (5.7)	15 (15.0)	
CRT	1 (1.0)	0 (0.0)	
Device duration, months, median (IQR)	36 (57)	72 (71.75)	**<0.001 ^b^**
Generator replacement, *n* (%)	7 (6.7)	21 (22.3)	**0.002 ^c^**
RBBB, *n* (%)	8 (7.7)	13 (13.0)	0.253 ^c^
QTc (Bazett), ms	434.30 ± 30.64	452.28 ± 42.30	**<0.001 ^a^**
Tp-e interval, ms, median (IQR)	103.9 (33.52)	122.4 (37.23)	**<0.001 ^b^**
Delayed intrinsicoid deflection, *n* (%)	76 (72.4)	94 (94.0)	**<0.001 ^c^**
Heart rate, bpm	70.23 ± 11.90	70.78 ± 14.05	0.762 ^a^
QRS duration, ms	111.25 ± 20.54	127.10 ± 34.82	**<0.001 ^a^**
Frontal QRS-T angle, °	−64.37 ± 93.54	−62.30 ± 99.35	0.878 ^a^
LVH, *n* (%)	4 (3.8)	11 (11.0)	**0.006 ^c^**
Delayed QRS transition (>V4), *n* (%)	48 (46.0)	74 (74.0)	**<0.001 ^c^**
ERS, mean ± SD	3.28 ± 1.39	4.79 ± 1.32	**<0.001 ^a^**
ERS category, *n* (%)			**<0.001 ^c^**
<4	75 (71.4)	9 (9.0)	
≥4	30 (28.6)	91 (91.0)	
Moderate-to-severe valve disease, *n* (%)	48 (49.0)	57 (57.0)	0.227
PR interval, ms	170.35 ± 29.76	182.47 ± 37.52	**0.017 ^a^**
LVEF, %	30.44 ± 7.30	29.79 ± 8.91	0.574 ^a^
LVEDD, mm	56.56 ± 7.77	58.24 ± 8.68	0.154 ^a^
LVESD, mm	43.69 ± 8.65	47.60 ± 9.66	0.175 ^a^
LA diameter, mm	43.14 ± 5.61	43.70 ± 7.01	0.098 ^a^
Ischemic cardiomyopathy, *n* (%)	17 (19.8)	29 (32.6)	0.054
Hypertension, *n* (%)	50 (63.3)	42 (51.9)	0.143
Diabetes, *n* (%)	39 (47.6)	24 (30.0)	**0.026**
Smoking, *n* (%)	37 (55.2)	29 (47.5)	0.385
Chronic renal failure, *n* (%)	12 (34.3)	9 (37.5)	0.800
COPD, *n* (%)	4 (18.2)	6 (35.3)	0.225
Cancer, *n* (%)	1 (5.6)	4 (33.3)	**0.046**
Cerebrovascular disease, *n* (%)	3 (15.0)	5 (35.7)	0.161
Beta-blocker, *n* (%)	96 (96.0)	92 (95.8)	0.953
ACEi/ARB, *n* (%)	80 (89.9)	75 (90.4)	0.917
MRA, *n* (%)	73 (84.9)	69 (89.6)	0.369
SGLT2 inhibitor, *n* (%)	43 (65.2)	36 (69.2)	0.640
ARNi, *n* (%)	15 (37.5)	14 (42.4)	0.669
GDMT, *n* (%)	77 (76.2)	65 (68.4)	0.221
Amiodarone, *n* (%)	12 (31.6)	23 (59.0)	0.016
Digoxin, *n* (%)	3 (9.7)	6 (22.2)	0.188

Data are presented as mean ± standard deviation (SD), median (interquartile range, IQR), or *n* (%). ^a^ Student *t*-test; ^b^ Mann–Whitney U test; ^c^ chi-square test. ACEi, angiotensin-converting enzyme inhibitor; ARB, angiotensin receptor blocker; ARNi, angiotensin receptor–neprilysin inhibitor; bpm, beats per minute; COPD, chronic obstructive pulmonary disease; CRT, cardiac resynchronization therapy; DR, dual-chamber; ERS, electrical risk score; GDMT, guideline-directed medical therapy; LA, left atrium; LVEDD, left ventricular end-diastolic diameter; LVEF, left ventricular ejection fraction; LVESD, left ventricular end-systolic diameter; LVH, left ventricular hypertrophy; MRA, mineralocorticoid receptor antagonist; QTc, corrected QT interval; RBBB, right bundle branch block; SGLT2, sodium-glucose cotransporter-2; Tp-e, Tpeak-Tend; VR, single-chamber. Bold *p*-values indicate statistical significance (*p* < 0.05).

**Table 2:** Comparison of biochemical values between the two groups.

Variable	Group 1 (*n* = 105)		Group 2 (*n* = 100)		*p*-Value
	*n*	Mean ± SD/Median (IQR)	*n*	Mean ± SD/Median (IQR)	
Creatinine, mg/dL	101	1.06 (0.32)	99	1.08 (0.32)	0.988 ^a^
GFR, mL/min/1.73 m^2^	97	70.84 ± 22.39	98	70.64 ± 21.87	0.779
Sodium, mmol/L	100	135.53 ± 19.05	99	137.46 ± 3.61	0.085
Potassium, mmol/L	95	4.40 ± 0.67	90	4.34 ± 0.51	0.437
Calcium, mg/dL	53	9.3 (0.8)	90	9.1 (0.8)	0.029 ^a^
Magnesium, mg/dL	65	2.0 (0.33)	63	2.0 (0.38)	0.395 ^a^
Albumin, g/dL	55	4.32 (0.37)	79	4.28 (0.28)	**0.005 ^a^**
Total cholesterol, mg/dL	98	170.37 ± 56.71	99	153.04 ± 42.75	0.108
HDL cholesterol, mg/dL	98	46.26 ± 13.19	99	40.73 ± 10.85	0.093
LDL cholesterol, mg/dL	97	94.96 ± 41.33	99	89.81 ± 34.82	0.453
Triglyceride, mg/dL	98	171.88 ± 183.83	99	112.47 ± 52.68	**0.013**
CRP, mg/L	55	2.95 (5.25)	79	2.30 (3.20)	0.090 ^a^
WBC, ×10^3^/µL	100	7.84 (2.03)	99	7.20 (0.27)	0.277 ^a^
Hemoglobin, g/dL	100	13.99 ± 1.74	99	13.41 ± 2.13	0.146
Platelet, ×10^3^/µL	100	235 (70)	99	209 (64)	0.122 ^a^
Neutrophil, ×10^3^/µL	98	4.36 (1.83)	99	4.03 (0.86)	0.674 ^a^
Lymphocyte, ×10^3^/µL	99	1.95 (0.59)	99	2.02 (0.30)	0.308 ^a^
BNP, pg/mL	41	479 (1331)	15	3587 (4014)	**0.034 ^a^**
Troponin, ng/L	63	8 (10)	74	6 (67)	0.443 ^a^
TSH, mIU/L	81	1.38 (1.00)	84	1.30 (2.00)	0.636 ^a^

Data are presented as mean ± standard deviation (SD) or median (interquartile range, IQR). ^a^ Mann–Whitney U test; other comparisons by Student *t*-test. BNP, B-type natriuretic peptide; CRP, C-reactive protein; GFR, glomerular filtration rate; HDL, high-density lipoprotein; LDL, low-density lipoprotein; TSH, thyroid-stimulating hormone; WBC, white blood cell. Bold *p*-values indicate statistical significance (*p* < 0.05).

**Table 3:** Receiver operating characteristic (ROC) curve analysis of the electrical risk score for discriminating patients with appropriate ICD therapy.

Parameter	Value (95% CI)
Area under the curve (AUC)	0.795 (0.733–0.857)
Optimal cut-off (Youden index)	3.5
Clinically applicable threshold	ERS ≥ 4
Sensitivity, % (*n*/N)	91.0 (83.8–95.2) (91/100)
Specificity, % (*n*/N)	71.4 (62.2–79.2) (75/105)
Positive predictive value, % (*n*/N)	75.2 (66.8–82.1) (91/121)
Negative predictive value, % (*n*/N)	89.3 (80.8–94.3) (75/84)

The optimal cut-off value was determined using the Youden index; because the ERS consists only of integers, a clinically applicable threshold of ERS ≥ 4 was adopted for subsequent analyses. CI, confidence interval; ERS, electrical risk score; *n*/N, number of correctly classified patients/total number of patients in the relevant outcome category.

**Table 4:** Univariable and multivariable logistic regression analyses of factors associated with appropriate ICD therapy.

Variable	Univariable OR (95% CI)	*p*-Value	Adjusted OR (95% CI)	*p*-Value
ERS	2.350 (1.423–3.881)	<0.001	2.189 (1.246–3.845)	**0.006**
LVEF, %	0.899 (0.809–0.998)	0.047	0.950 (0.827–1.092)	0.471
Device duration, months	1.004 (0.987–1.021)	0.672	1.011 (0.986–1.036)	0.399
Age, years	0.960 (0.900–1.024)	0.218	0.959 (0.883–1.041)	0.314
BNP, pg/mL	1.000 (1.000–1.000)	0.420	1.000 (1.000–1.000)	0.438
Cardiomyopathy (ischemic)	1.200 (0.301–4.786)	0.796	1.130 (0.146–8.764)	0.907
Sex (male)	1.048 (0.234–4.691)	0.951	1.144 (0.167–7.853)	0.891

Model performance: model χ^2^ = 21.394, *p* = 0.006; Cox & Snell R^2^ = 0.431; Nagelkerke R^2^ = 0.604; McFadden R^2^ = 0.451. BNP, B-type natriuretic peptide; CI, confidence interval; ERS, electrical risk score; LVEF, left ventricular ejection fraction; OR, odds ratio. Bold values indicate statistical significance (*p* < 0.05).

**Table 5:** Univariable and multivariable logistic regression analyses of the association between the electrical risk score components and appropriate ICD therapy.

ERS Component (Present)	Group 1 (*n* = 105)	Group 2 (*n* = 100)	Total (*n* = 205)	OR (95% CI)	*p*-Value	Adjusted OR (95% CI)	*p*-Value
Heart rate > 75 bpm	31 (29.5)	33 (33.0)	64 (31.2)	1.18 (0.65–2.13)	0.59	1.03 (0.50–2.10)	0.94
Prolonged QTc (>450/460 ms)	27 (25.7)	48 (48.0)	75 (36.6)	2.67 (1.49–4.85)	0.001	2.08 (1.02–4.29)	**0.045**
Prolonged Tp-e (>89 ms)	74 (70.5)	90 (90.0)	164 (80.0)	3.77 (1.79–8.57)	<0.001	1.98 (0.83–4.99)	0.13
QRS duration > 110 ms	38 (36.2)	65 (65.0)	103 (50.2)	3.27 (1.86–5.85)	<0.001	2.21 (1.09–4.53)	**0.029**
Wide QRS-T angle (>90°)	46 (43.8)	59 (59.0)	105 (51.2)	1.85 (1.06–3.23)	0.03	1.20 (0.61–2.31)	0.60
LVH	4 (3.8)	11 (11.0)	15 (7.3)	3.12 (1.03–11.6)	0.059	7.93 (2.03–40.1)	**0.006**
Delayed QRS transition (≥V5)	48 (46.0)	74 (74.0)	122 (59.5)	3.38 (1.89–6.16)	<0.001	4.33 (2.17–9.01)	**<0.001**
Delayed intrinsicoid deflection	76 (72.4)	94 (94.0)	170 (82.9)	5.98 (2.51–16.6)	<0.001	NC	—

Data are presented as *n* (%). aOR, adjusted odds ratio; CI, confidence interval; ERS, electrical risk score; LVH, left ventricular hypertrophy; OR, odds ratio; QTc, corrected QT interval; Tp-e, Tpeak-Tend interval. NC, not calculable (the adjusted estimate did not converge). The wide confidence interval for LVH reflects the small number of events and should be interpreted with caution. Bold values indicate statistical significance (*p* < 0.05).

**Table 6:** Internal validation and optimism correction of the multivariable model and the standalone electrical risk score.

Metric	Multivariable Model (ERS + Covariates)	Univariable Model (ERS Alone)
Apparent AUC	0.840	0.795
10-fold CV AUC (mean ± SD)	0.822 ± 0.103	0.790 ± 0.088
Model optimism (bootstrap)	0.019	0.0000
Optimism-corrected AUC	0.821	0.795
Bootstrap 95% CI for AUC	0.790–0.899	0.733–0.857
Apparent Brier score	0.162	0.198
Optimism-corrected Brier score	0.176	0.198

Internal validation was performed using 10-fold cross-validation and bootstrap resampling with 2000 replications. The multivariable model included the electrical risk score (ERS) together with age, sex, left ventricular ejection fraction, cardiomyopathy etiology, B-type natriuretic peptide, and device duration. Model optimism represents the difference between apparent and internally validated performance; the optimism-corrected AUC and Brier score provide bias-adjusted estimates of discrimination and calibration, respectively. AUC, area under the curve; CI, confidence interval; CV, cross-validation; ERS, electrical risk score; SD, standard deviation.

**Table 7:** Comparison of the original six-component ERS and the modified eight-component ERS for discriminating patients with appropriate ICD therapy.

Metric/Parameter	Original Six- Component ERS	Modified Eight- Component ERS	Difference/Statistic	*p*-Value
Area under the curve (AUC)	0.731 (0.665–0.798)	**0.795 (0.733–0.857)**	ΔAUC = +0.064	0.0012 ^a^
Adjusted OR (95% CI)	2.571 (1.779–3.715)	2.189 (1.246–3.845)	—	**<0.001**
Model AIC	199.25	**193.39**	ΔAIC = −5.86	Superior fit
Continuous NRI	Reference	**0.629**	Events: 0.303; non-events: 0.326	**<0.001**
IDI	Reference	**0.056**	Absolute gain: +5.6%	**0.002**

^a^ DeLong test for paired ROC curves. AIC, Akaike Information Criterion; CI, confidence interval; ERS, electrical risk score; IDI, Integrated Discrimination Improvement; NRI, Net Reclassification Improvement; OR, odds ratio. The original six-component ERS included heart rate, QRS transition, frontal QRS-T angle, QTc interval, Tpeak–Tend interval, and left ventricular hypertrophy; the modified eight-component ERS additionally included QRS duration (>110 ms) and delayed intrinsicoid deflection as depolarization parameters. Adjusted odds ratios were derived from multivariable logistic regression models adjusted for age, sex, left ventricular ejection fraction, cardiomyopathy etiology, and device duration. Bold values indicate statistical significance (*p* < 0.05).

## Data Availability

The data presented in this study are available on request from the corresponding author. The data are not publicly available due to privacy and ethical restrictions.

## Abbreviations

The following abbreviations are used in this manuscript:

AF	atrial fibrillation
ARNi	angiotensin receptor–neprilysin inhibitor
ATP	antitachycardia pacing
BNP	B-type natriuretic peptide
bpm	beats per minute
CIED	cardiac implantable electronic device
CL	cycle length
CRP	C-reactive protein
CRT-D	cardiac resynchronization therapy–defibrillator
ECG	electrocardiogram
ERS	electrical risk score
GDMT	guideline-directed medical therapy
GFR	glomerular filtration rate
HDL	high-density lipoprotein
HF	heart failure
HFrEF	heart failure with reduced ejection fraction
ICD	implantable cardioverter-defibrillator
LBBB	left bundle branch block
LDL	low-density lipoprotein
LVEDD	left ventricular end-diastolic diameter
LVEF	left ventricular ejection fraction
LVESD	left ventricular end-systolic diameter
LVH	left ventricular hypertrophy
MRA	mineralocorticoid receptor antagonist
MRI	magnetic resonance imaging
NYHA	New York Heart Association
OR	odds ratio
QTc	corrected QT interval
RBBB	right bundle branch block
ROC	receiver operating characteristic
SCD	sudden cardiac death
SGLT2	sodium-glucose cotransporter-2
Tp-e	Tpeak-Tend interval
TSH	thyroid-stimulating hormone
VF	ventricular fibrillation
VT	ventricular tachycardia
WBC	white blood cell
WCD	wearable cardioverter-defibrillator
